# Novel and unscrutinized immune entities of the zebrafish gut

**DOI:** 10.1002/1873-3468.70161

**Published:** 2025-09-12

**Authors:** Audrey Inge Schytz Andersen‐Civil, Marine Mantel, Julia Soh, Serge Mostowy, Sylvia Brugman, Gilles Claude Vanwalleghem

**Affiliations:** ^1^ Department of Molecular Biology and Genetics Aarhus University Aarhus C Denmark; ^2^ Danish Research Institute of Translational Neuroscience – DANDRITE, Nordic‐EMBL Partnership for Molecular Medicine Aarhus Denmark; ^3^ Department of Infection Biology London School of Hygiene & Tropical Medicine London UK; ^4^ Host Microbe Interactomics, Animal Sciences Group Wageningen University and Research Wageningen The Netherlands

**Keywords:** gut immunity, host–pathogen interactions, microbiota, translational research, zebrafish

## Abstract

The zebrafish model offers a unique opportunity to study gut immunity due to its diverse applicability within several fields of research, combined with an evolutionarily conserved immune system, transparency, and genetic tractability. This review highlights recent advances in understudied immune cell types in the zebrafish gut, emphasizing their potential to illuminate immune processes of the vertebrate immune system. The biological function of the gut is highly conserved in zebrafish, which makes them a relevant model to study intestinal immune cells with advanced molecular and imaging techniques that enable *in vivo* visualization of immune mechanisms and cell trajectories. Rodent and pig models have successfully contributed to our understanding of many aspects of the immune system, while zebrafish have so far been underestimated in their potential role in furthering our knowledge in this field. We suggest how future study directions can help elucidate the complex nature of gut immunity and highlight similarities between mammalian and zebrafish immune systems. Provided that immune cell functions are conserved, zebrafish can offer great opportunities for translational studies and have an important impact in improving human health.

## Graphical abstract

Understudied cells of the zebrafish immune system include bona fide immune cells and epithelial (‐derived) cells with immune functions. Research focusing on zebrafish cells which demonstrate similarities to mammalian immune cell counterparts may help us understand the pathologies in which they are implicated. Currently available and advanced tools make zebrafish a promising model for advancing our understanding of gut immunity. Figure created in BioRender. Andersen, I. (2025) https://BioRender.com/6wf6igb.

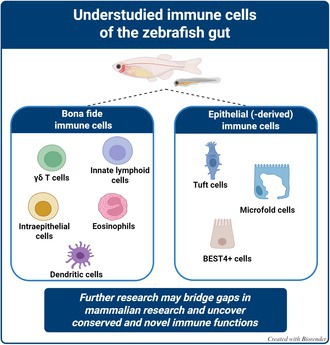

## Abbreviations

**cDC**, conventional dendritic cells

**dpf**, days postfertilization

**IELs**, intraepithelial lymphocytes

**IgE**, immunoglobulin E

**ILCs**, innate lymphoid cells

***lck***, tyrosine kinase

**M cells**, microfold cells

**NK**, natural killer cell

**NLRs**, NOD‐like receptors

**PAMPs**, pathogen‐associated molecular patterns

**pDC**, plasmacytoid dendritic cells

**PRRs**, pattern recognition receptors

**SEM**, scanning electron microscopy

**TCR**, T‐cell receptors

**TEM**, transmission electron microscopy

**Th**, T helper cell

**TLRs**, toll‐like receptors

**Treg**, T regulatory

Since the use of zebrafish for research was pioneered by Streisinger et al. [1], the vast applications of this promising model have been exponentially increasing. Transparency, high fecundity, a fully sequenced genome, and easy genomic manipulation make them an attractive model for many research areas. However, these advantageous features have not yet been fully exploited in the field of immunology, since zebrafish have not been used as a model for as many years as, for example, rodent models. Potential unexplored areas of immunological research could be enlightened by using cutting‐edge techniques such as single‐cell RNA sequencing and advanced imaging tools such as light‐sheet microscopy. Using transgenic reporter lines of specific immune factors in zebrafish, the localization and function of specific immune cells can be tracked *in vivo*, which is not as feasible in other vertebrate animal models.

There are several reviews on zebrafish hematopoietic stem cells, innate immune cells, and host–pathogen interactions [2, 3]. However, in this review, we focus on understudied immune cells in zebrafish and whether recently discovered immune cells from mammalian models have been identified and characterized in the zebrafish. Our aim is to offer an overview of parts of the immune system related to gut health that remain largely unknown, or which have only been recently discovered in the zebrafish; we also provide future directions that could aid the identification of their functions.

**Fig. 1:**
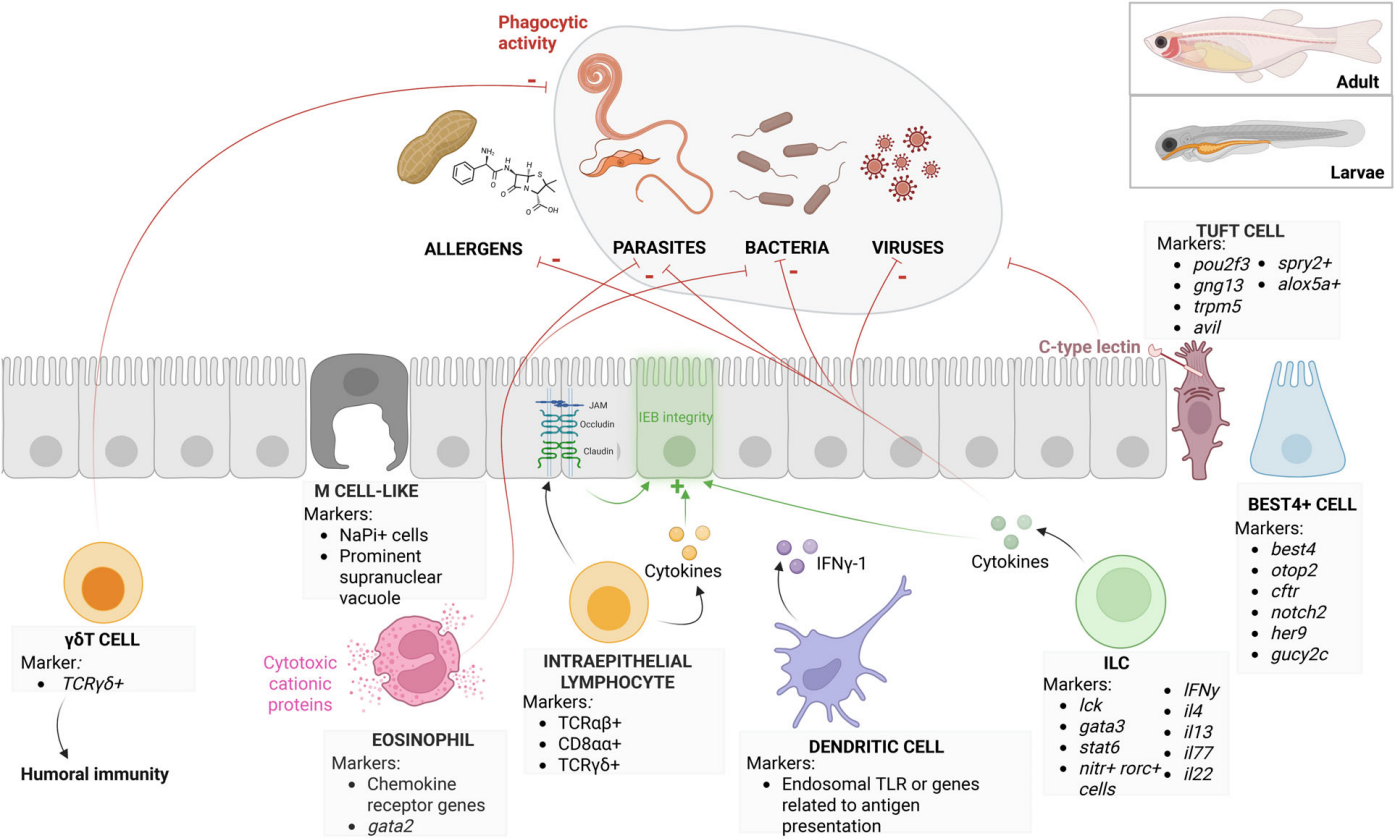
**Markers and functions of understudied immune cells in the zebrafish gut.** Understudied immune cell types in the zebrafish gut, along with their main associated markers and roles in zebrafish gut immunity. IFN, interferon; ILC, innate lymphoid cells; IL, interleukin; TLR, Toll‐like receptor. *Figure* *created in BioRender*. Andersen, I. (2025) https://BioRender.com/um7scu9.

The immune system is highly conserved between zebrafish and mammals. As shown in Fig. 1, the zebrafish immune system shares many markers and key mechanisms with that of mammals, including the recognition of pathogen‐associated molecular patterns (PAMPs) in microbes through pattern recognition receptors (PRRs), cytokine production, complement activation, and the stimulation of cellular effectors [4]. These similarities highlight the relevance of zebrafish as a model for elucidating the functions of immune factors that may play a crucial role in human diseases.

Gut health has gained significant attention in recent years, and it has become clear that it plays a central role in overall health. The general anatomical architecture of the gut of zebrafish is conserved, and many of the common immune cells described in mammals are also present. Thus, immune cells such as macrophages and neutrophils are well described in zebrafish and their functions are similar to mammals. Major innate immune signaling pathways including the MyD88‐dependent pathway, the JAK–STAT pathway, NOD‐like receptors (NLRs) and inflammasome pathways, and effector mechanisms such as activation of Toll‐like receptors (TLRs) and transcription factors such as NF‐κB, are highly conserved between zebrafish and mammals [5, 6, 7, 8]. Genomic approaches have also demonstrated substantial common regulation and conservation of gene expression in intestinal epithelial cells [9]. Zebrafish adults also harbor adaptive immune cells like B and T cells, which are functionally equivalent to the mammalian B and T cells and have been comprehensively reviewed elsewhere [10, 11]. However, some discrepancies exist as certain immune components are seemingly missing in zebrafish. For example, lymph nodes, Peyer's Patches, Paneth cells, and M cells have not been reported in zebrafish. Additionally, zebrafish do not develop a functional adaptive immune system until later in life. While T cells have been shown to leave the thymus at 10 days postfertilization, full functionality of these T cells has not been demonstrated. In general, it is believed that the adaptive immune system of zebrafish is fully mature between the fourth and sixth weeks of development, although this early egress of T cells is intriguing [12, 13, 14]. This delay limits the study of the intricate interactions between the innate and adaptive immune systems before these time points. On the other hand, zebrafish heavily rely on their innate immune system during early life stages and, compared to mammals, it is likely more adapted to an environment in which mucosal defenses within the skin, gills, and intestinal tract are highly crucial for survival [15, 16]. This allows for targeted research aiming at unraveling the role of isolated innate immune factors.

Zebrafish models of gut inflammation provide a valuable platform to understand how the immune system responds to and mitigates inflammation. As listed in Table 1, there are many advantages of using zebrafish to study gut immunity. Given the significant burden of intestinal inflammatory diseases today [17], these models are particularly important for advancing our knowledge in this field. Modeling such responses can enable the understanding of the role of specific immune factors during inflammation in specific settings which cannot easily be done in, for example, rodent models. For example, the intestinal environment of zebrafish can be manipulated by immersion, and while maintaining germ‐free zebrafish beyond 8 days can be challenging due to feeding requirements, germ‐free larvae can be maintained with relative ease [18]. This allows researchers to study the effect of the gut microbiome on gut immunity compared to germ‐free fish. Host–pathogen interactions can also be assessed to evaluate the impact of specific pathogens with/without the impact of the microbiome. Thus, the combination of advanced molecular techniques and relevant experimental designs makes zebrafish a promising model to study gut immunity.

**Table 1:** Advantages and limitations of using zebrafish models to study gut immunity.

**Advantages**
Conserved immune system and intestinal function compared to mammals ° More than 70% of human genes have zebrafish orthologs Efficient genetic manipulation enables generation of transgenic lines ° Reporter lines of specific markers ° Disease models Controlled environment ° Germ‐free raised zebrafish ° Inoculation of pathogens by immersion or micro‐gavage ° Drug‐induced intestinal inflammation by immersion or micro‐gavage ° Controlled dietary interventions Optical transparency ° Imaging of internal organs *in vivo* ° Recording of gut motility *in vivo* ° Transgenic reporter lines with, for example, specific tissue‐restricted gene expression and fluorescently marked cell types and/or immune factors Study of the innate immune system in isolation in zebrafish larvae <6 weeks postfertilization High‐throughput screening of potential drugs Rapid *ex vivo* development High fecundity Cost efficient Study of single cell to whole organism outcome Unlike organoids, there is a complete immune response
**Disadvantages**
Anatomical limitations ° Simplified intestinal anatomy ° Absence of, for example, a submucosal plexus, intestinal crypts, intestinal Paneth cells, and Peyer's patches Lack of gastric glands in the intestinal bulb Lack of commercial antibodies for cell surface markers and proteins compared to mammals Body temperature differences between fish (~28 °C) vs mammals (~37 °C) The microbiome mainly consists of Proteobacteria and Fusobacteria as opposed to Bacteroidetes and Firmicutes in mammals Outbred vs inbred (as compared to mice) Teleost duplication of some genes
**References**
[18, 75, 76, 77, 78, 79, 80]

In this review, we focus on the least scrutinized cells of the immune system in zebrafish that have also been reported in mouse and human studies, including bona fide immune cells (e.g., innate lymphoid cells, intraepithelial lymphocytes, eosinophils, γδ T cells, and dendritic cells) and epithelial or epithelial‐derived cells with immune functions (e.g., tuft cells, BEST4+ cells, and M‐cell‐like enterocytes). Providing a better understanding of these cells using a zebrafish model that demonstrates similarities to their mammalian counterparts will potentially help us understand human diseases in which these cells are implicated.

## Bona fide immune cells

### Innate lymphoid cells

Innate lymphoid cells (ILCs) represent some of the most recently discovered cells of the innate immune system and are commonly categorized as ILC1, ILC2, and ILC3 cells [19, 20]. They are considered the innate counterparts of the adaptive T helper 1 (Th1), Th2, and Th17 immune responses and have profound impact on mucosal immunity, including gut homeostasis [21]. Their main function is to secrete effector cytokines and thereby regulate other immune cells of the innate and adaptive immune system. ILCs also contribute directly to gut barrier integrity by secreting IL‐22 and IL‐26 [22, 23]. Depending on a given inducer of inflammation, the immune system establishes a specialized response towards different pathogens. Intracellular bacteria and viruses primarily activate ILC1, which helps eliminate these pathogens. Parasites and allergens stimulate ILC2, while extracellular bacteria activate ILC3. In addition to fighting infections, ILC3 plays a key role in maintaining the intestinal epithelial barrier. Beyond their classification into distinct subsets, ILCs exhibit a remarkable degree of plasticity, allowing them to adapt to changing microenvironmental cues [24]. There are still many open questions regarding ILC plasticity, particularly the mechanisms that drive their phenotypic and functional transitions. While studies have demonstrated that ILCs can adapt to different microenvironmental cues by shifting between subsets, the molecular pathways and transcriptional networks governing this process remain largely unclear. A key question is whether these conversions are fully reversible or not. Another major unknown is the interaction of ILCs with other immune cells. While it is well established that ILCs respond to cytokine signals from various immune cells, the bidirectional nature of these interactions remains poorly characterized. Addressing these questions will be essential to fully understand the role of ILCs in immune regulation and their potential as therapeutic targets.

So far, only two recent papers have revealed the presence of ILC‐like cells in larvae and adult zebrafish by single‐cell RNA sequencing (Hernández et al., 2018; Peng et al., 2023). The induction of lymphocyte‐specific protein tyrosine kinase (*lck*), a shared marker for ILC cells and T cells, was significantly increased in the gut of germ‐free zebrafish at 5 days postfertilization (dpf) when exposed to graphene oxide and butyrate [26]. Gene expression profiling in whole larvae revealed upregulation of the transcription factor genes *gata3* and *stat6*, along with the cytokine‐encoding genes, *il4* and *il13*, suggesting the presence of ILC2‐like cells in zebrafish larvae. However, assessing ILCs in larvae is limited by the absence of functional adaptive immunity and may not entirely correlate with immune responses observed in a more developed/mature organism.

The presence of lymphocytes corresponding to mammalian ILC1, ILC2, and ILC3 lineages was also assessed in the gut of wild‐type and *rag1*‐deficient zebrafish. Rag1 and Rag2‐deficient mice with no mature T or B cells have previously been used to study ILCs, and, conveniently, T and B lymphocytes are also lacking in *rag1*‐deficient zebrafish [25, 27]. Challenging zebrafish with immunogens by intraperitoneal injections led to the upregulation of ILC‐related genes [25]. Stimulation with formalin‐inactivated bacteria (*Vibrio anguillarum* strain 1669) induced the expression of T_H_1/ILC1 cytokines, such as *ifng1‐1* and *ifng1‐2*, as well as T_H_17/ILC3 cytokines *il17a/f3* and *il22* in both wild‐type and *rag1* ^−/−^ zebrafish. On the other hand, treatment with helminth extracts (lyophilized Anisakis simplex larvae) led to the expression of T_H_2/ILC2 cytokines *il4* and *il13*. To specifically identify ILCs, which represent <5% of lymphocytes of the intestinal barrier and express *lck*, the transgenic zebrafish line *Tg(lck:EGFP) rag1* ^*−/−*^ was used. This allowed for the identification of *nitr* ^+^ *rorc* ^+^ cells expressing *il22* and *tnfa* in zebrafish challenged with bacteria, suggesting the presence of ILC3‐like cells [25]. Consistent with this, the identification of *nitr* ^+^ *rorc* ^+^ cells expressing *il13* and *gata3* in zebrafish injected with helminth extracts suggests the presence of ILC2‐like cells. Finally, PBS controls harbored cells that exclusively expressed *ifng1‐1* but not granzymes or NK‐lysins, which could be considered ILC1‐like cells in zebrafish [25].

So far, research on zebrafish has provided valuable insights into the presence and potential function of ILC‐like cells and has demonstrated that zebrafish ILC‐like cells respond to bacterial and helminth challenges by upregulating lineage‐specific cytokines, mirroring mammalian ILC responses. However, the precise function of these cells in zebrafish immunity remains largely unexplored. In addition, the upregulation of ILC‐related markers does not serve as definitive evidence for ILC presence or activation and is thus described as ILC‐like cells for the time being. Given the genetic tractability and transparency of zebrafish larvae, this model provides a unique opportunity to further dissect the mechanisms of ILC differentiation, plasticity, and interactions with other immune cells, potentially uncovering evolutionary conserved and species‐specific aspects of ILC biology.

### Intraepithelial lymphocytes

Intraepithelial lymphocytes (IELs) in the intestine constitute a critical first line of defense against the external environment and play a key role in maintaining the integrity of the mucosal barrier. IELs include conventional T‐cell receptors (TCR) αβ + and unconventional CD8αα + lymphocytes, which can be either TCR𝛾δ + or TCRαβ+ [28]. TCR𝛾δ + IELs represent the largest population of gut IELs in mice. In mammals, gut IELs, particularly the highly motile γδ IELs, are essential for limiting pathogen translocation, preserving epithelial tight junctions, and producing antimicrobial peptides such as RegIIIγ. The function of individual IEL subsets remains largely unknown, primarily due to the lack of tools that allow for the selective targeting of these subsets. Another major question in the IEL field concerns the balance between stability and plasticity of IEL subsets under acute versus chronic inflammatory conditions. While some evidence suggests that IELs may exhibit a degree of plasticity in response to inflammatory cues, how this plasticity translates into functional changes over time and in different immune contexts remains unclear.

Despite their importance, IELs remain largely understudied in zebrafish. A recent study demonstrated that probiotic and postbiotic treatments not only increase the proportion of intraepithelial leukocytes in the zebrafish gut, but also enhance barrier function, suggesting a role for these cells in maintaining barrier integrity through cytokine production and the upregulation of junctional molecule expression [29]. However, it is important to note that in this study, the term ‘intraepithelial leukocytes’ refers to immune cells within the epithelium in a broad sense, as no specific labeling was used to confirm their lymphoid identity. Thus, while intraepithelial leukocytes have been identified in zebrafish, true intraepithelial lymphocytes—as defined by lineage‐specific markers—have not yet been clearly characterized. Further research is needed to define the nature and function of these cells in zebrafish, which may reveal conserved principles of mucosal immunity across vertebrate species.

### Eosinophils

Eosinophils are granulocytic leukocytes primarily involved in host protection against parasitic infections. Beyond this classical role, they exhibit additional functions, such as antibacterial and antifungal activities, regulation of other immune cell populations, maintenance of tissue homeostasis, and promotion of liver and muscle regeneration [30]. In adult hematopoiesis, eosinophils originate from eosinophil lineage‐committed progenitors, which, in humans, arise from common myeloid progenitors, and in mice, from granulocyte/macrophage progenitors. In zebrafish, the specific pathways of eosinophil differentiation remain less well‐defined but are thought to share similarities with mammalian systems. This is the case of Cebp1, a functional ortholog of human C/EBPεP27, in repressing eosinophilopoiesis [31].

Under homeostatic conditions, eosinophils are relatively rare in the blood and most tissues, including the gastrointestinal tract. In uninfected fish, eosinophils are sparsely distributed along the base of the intestinal lamina propria. However, inflammatory stimuli or infections can significantly increase eosinophil numbers, a response conserved in zebrafish. Notably, zebrafish eosinophils react to parasitic infection, particularly helminths, in a manner comparable to their mammalian counterparts. This includes degranulation of cytotoxic cationic proteins to combat parasites. Interestingly, three ribonucleases with cationic and bactericidal properties have been identified in zebrafish eosinophils, highlighting their potential antimicrobial role. Additionally, zebrafish eosinophils express chemokine receptor genes, including homologs for mammalian receptors, suggesting functional conservation across species [32].

Overall, eosinophils play diverse roles beyond parasite defense, including antimicrobial activity, immune regulation, and tissue homeostasis. While their differentiation pathways in zebrafish remain less defined, the functional parallels with mammals, particularly in response to infections, underscore zebrafish as a promising model for studying eosinophil and immune mechanisms. Reporter lines with specifically labeled cells of the eosinophil lineage from early life through adulthood exist and can be used to explore the spatial and temporal behavior of these cells; such lines include the TgKI (embp‐tdTomato, cryaa:EGFP) zebrafish line, which enables *in vivo* visualization and flow cytometric analysis of eosinophils throughout development [31, 32, 33].

### γδ T cells

γδ T cells are a unique IEL subpopulation discovered in 1986, with high proportions found in peripheral tissues [34]. These T cells are rare in circulation but enriched in the gut. γδ T cells diverge developmentally from conventional αβ T cells, and their putative TCRγδ ligands exhibit remarkable diversity in sources, structures, and binding mechanisms. Additionally, they express several natural killer (NK) cell‐associated receptors and can respond to signals in a TCR‐independent manner. As a result, γδ T cells do not adhere to the classical rules of T‐cell biology [35]. These lymphocytes express a T‐cell receptor composed of γ and δ chains, which they use as PRRs to recognize PAMPs. Upon activation, γδ T cells rapidly produce large amounts of cytokines. They are involved in immune surveillance activities, including direct killing of infected cells, recruitment of neutrophils, activation of phagocytes, and induction of granuloma formation. A distinctive feature of γδ T cells is their ability to exhibit memory phenotypes and adaptive‐like responses during certain infections, positioning them as a bridge between innate and adaptive immunity [36]. Importantly, γδ T cells play a crucial role in tumor immunity through their ability to lyse tumor cells, enhance CD8^+^ αβ T‐cell memory, and promote wound resolution. Their TCRs recognize a broad range of tumor targets and, unlike αβ T cells, they are not MHC‐restricted, allowing for allogeneic transfusion with minimal risk of graft‐versus‐host disease. However, the tumor microenvironment suppresses γδ T‐cell function through various mechanisms, contributing to immune evasion. A key challenge in the field is to decipher the specific mechanisms by which the tumor microenvironment suppresses γδ T‐cell function [37].

Relatively few studies have investigated the presence and functions of these cells in the zebrafish model. However, γδ T cells have been identified in zebrafish using methods such as flow cytometry, transmission electron microscopy (TEM) and scanning electron microscopy (SEM). Zebrafish γδ T cells exhibit phenotypic and morphological similarities to their mammalian counterparts. Notably, in adult zebrafish, γδ T cells demonstrate potent phagocytic activity, act as antigen‐presenting cells, and participate in the activation of systemic adaptive humoral immunity [38]. This functional overlap highlights the role of zebrafish γδ T in linking innate and adaptive immune responses, similar to mammals.

Zebrafish thus represent an emerging model for investigating γδ T‐cell biology and γδ T‐cell‐mediated diseases. The molecular and functional conservation of γδ T cells between zebrafish and mammals, along with their crucial role in immune regulation, infectious diseases, and autoimmune disorders, underscores the relevance of this model. Thus, further research is needed to elucidate the mechanisms of action of these cells and to conduct cross‐species investigations.

### Dendritic cells

Dendritic cells are antigen‐presenting cells that play a central role in orchestrating immune responses towards harmful stimuli. They are found at mucosal barriers, such as the gut, where they perform receptor‐mediated phagocytosis, and migrate to the draining lymph nodes to activate the adaptive immune response by inducing the polarization of naïve T cells [39]. Dendritic cells were first described in the 1970s in mice [40]; however, they were only discovered in zebrafish 40 years later by Lugo‐Villarino et al. in various adult tissues including the gut [41]. Subsequent reporter lines combined with morphologic and molecular analyses have also demonstrated their (admittedly scarce) presence in intestinal tissues [42, 43].

Similar to mammals, adult zebrafish were recently found to harbor two main dendritic cell populations by single‐cell transcriptome profiling, that is, conventional dendritic‐like cells (cDC) expressing genes related to antigen presentation, and the plasmacytoid dendritic‐like cells (pDC) defined by their unique expression of endosomal TLRs and massive production of type 1 interferon [43]. Another study also reported that cells with morphological features of monocyte‐derived dendritic cells with long protrusions were identified in zebrafish larvae during intestinal inflammation [44].

Although the presence of dendritic cells in the gut of zebrafish has been described, further research is needed to fully understand the development and functional specialization of individual dendritic cell subpopulations, as well as resolve the uncertainty regarding where they migrate upon activation. Their role in context‐dependent disease settings may potentially pave the way for the use of new applications of the zebrafish model to study diseases related to these cells.

## Epithelial‐derived cells with immune functions

### Tuft cells

Tuft cells are chemosensory cells that were discovered in the 1950s; although they have attracted greater interest in many research fields in recent years, their various functions in immune regulation remain largely unknown [45, 46]. Tuft cells primarily reside within the mucosal tissues of the respiratory tract and the gut and have also been identified in the thymus [47]. They are part of the secretory cell lineages of the intestinal epithelium together with goblet, enteroendocrine, and Paneth cells. They are known to sense luminal cues, including microbial metabolites, and respond by producing cytokines such as IL‐25. Tuft cell hyperplasia within the gut has been reported during parasite infection and has been found to play a fundamental role in the activation of the type 2 immune response in conjunction with ILC2 and Th2 cells. The secretion of IL‐25 by tuft cells is crucial for efficient worm expulsion, which activates IL‐13 secretion by ILC2 and promotes tuft and goblet cell hyperplasia [48, 49].

Tuft cells were first reported in zebrafish by Willms et al. where they performed single‐cell transcriptional profiles of the digestive tract in 6 dpf zebrafish larvae [50]. They identified cells that were enriched for expression of markers highly associated with intestinal tuft cells, including the *pou2f3* master regulator, *Gng13*, *Trpm5*, and *Avil*. Another paper confirmed the presence of tuft cells in adult zebrafish by transcriptional profiling and identified cells expressing *pou2f3* ^+^, *spry2* ^+^, and *alox5a* ^+^ [51]. They distinguished two tuft cell subsets distinguished by expression of *si*:*dkey‐61f9*.*1*, which encodes a C‐type lectin domain‐containing protein that exhibits homology to the immunoglobulin E (IgE) receptor Fcer2. TEM of the posterior intestinal epithelium in adult zebrafish also revealed a cell type with morphological characteristics of tuft cells, with an apical tuft protruding through the epithelial brush border [51].

With only two recent reports describing the presence of tuft cells in zebrafish, this area of research contains an important knowledge gap that needs further exploration. Studies involving parasitic infections in zebrafish may pave the way for identifying key immune functions of tuft cells that may correspond to functions described in mammalian models. Furthermore, tuft cells remain a rare cell of the intestinal lining, which may demand novel approaches to identify their potential roles in different disease settings—settings which could be modeled in zebrafish.

### BEST4 ^+^ cells

Absorptive cells, also known as enterocytes, represent the predominant cell type of the intestinal epithelium. Next to their primary role in digestion of nutrients, enterocytes are also involved in several immune processes, such as presenting processed antigens to T cells [52]. Recently, a new lineage of mature absorptive cells in the human intestinal epithelium has been identified as BEST4^+^ cells, which are characterized by the expression of Bestrophin 4 (BEST4) and marker genes such as *otop2*, *ca7*, *guca2a*, *guca2b*, and *spib* [53].

Although the role of BEST4^+^ cells in gut health remains unclear, they are believed to play a role in maintaining intestinal homeostasis since excess BEST4 activity may lead to the progression and metastasis of colorectal cancer [51, 54]. Emerging evidence also suggests that BEST4^+^ cells may respond to microbial or immune‐derived signals to modulate intestinal homeostasis. BEST4^+^ cells have not been found in mice, but have been shown to be present in the intestinal epithelium of macaque, pig, rabbits, and zebrafish [53]. Gene expression data from conventional adult zebrafish revealed the presence of BEST4^+^ cells expressing *best4*, *otop2*, and *cftr* [51]. Sur et al. describe strong molecular similarities between human and zebrafish BEST4^+^ cells by identifying specific shared markers such as *best4*, *otop2*, *cftr*, *notch2*, *her9*, and *gucy2c* [55]. Thus, future studies in the zebrafish model could further our understanding of the role of BEST4^+^ cells in a variety of disease models.

### Microfold cells

Microfold cells, also known as M cells, are specialized enterocytes that are localized in Peyer's patches within the intestinal epithelial layer. They take up and deliver antigens to antigen‐presenting cells, for example, dendritic cells and lymphocytes, and play a fundamental role in mucosal immune surveillance and initiation of inflammatory responses. These cells are widely described in mammalian models, and similar cells have also been described in cyprinids [56], where they were proposed to participate in immune surveillance in the gut. Additionally, M‐cell‐like cells have been reported in the gills of rainbow trout [57]. In zebrafish, analogous cells have been identified and studied primarily for their digestive functions. However, their potential role in immune responses and antigen sampling has been largely overlooked in the literature, despite strong morphological and functional similarities with mammalian M cells [58]. However, a subtype of enterocytes with M‐cell‐like characteristics have been described by Wallace et al. [59]. They describe NaPi+ enterocytes in both larvae and adult zebrafish that contain a prominent supranuclear vacuole in which pinocytosed luminal contents can be stored. These have been suggested to function as antigen‐presenting cells that may be analogous to the M cells of the adult mammalian intestine. Histochemical assessment showed that most NaPi+ cells within the posterior segment of the mid intestine are specialized enterocytes [59]. A more recent study investigated the translocation of bacteria and nanoparticles across the intestinal epithelium in adult zebrafish; the translocated cells and particles were rapidly taken up and transported to the liver and spleen. The study also discusses the potential presence of cells with M‐cell‐like features that may have the ability to sample or even deliver particles such as nanoparticles or whole microorganisms to associated antigen‐presenting cells [60]. Specialized cells with M‐cell‐like features are also sometimes mentioned as lysosome‐rich enterocytes.

Although M cells have not yet been clearly identified in zebrafish, enterocytes with absorptive, antigen‐presenting, and M‐cell‐like features have been described. Thus, further studies are needed to validate and explore whether specific cell subtypes, such as NAPi+ cells, may serve as a potential counterpart for mammalian M cells.

## Discussion and future directions

The field of immunology is complex, and although many cell types have been extensively investigated, researchers frequently find new cellular mechanisms that are highly context dependent. Immune cells can have differing functions depending on disease state, age, time of the day, tissues, etc., which challenges their study. However, several components of the immune system, especially the innate immune system, are conserved across many vertebrates, such as mammals and zebrafish [61, 62, 63]. Classically used models, such as mice and pigs, have helped further our understanding of many immune processes, but these models also have their own limitations (e.g., higher costs, laborious experimental set‐ups, and species‐specific immunity), and thus, the zebrafish can be used as a complementary model. One of the key features of zebrafish is their transparency during early life stages, which allows researchers to use reporter lines to track specific immune cells and/or pathogens by fluorescence microscopy. Although *in vivo* live tracking of immune cells is more commonly applied in zebrafish larvae, it is also used in adults [64]. Moreover, as highlighted throughout this review, genetic manipulation to study the role of these cell types *in vivo*; for example, knockout and reporter zebrafish, as well as omics approaches to characterize cells (including RNAseq, CHIPseq), can provide invaluable insights into immune cell functions. Generation of cell‐type‐specific reporters is needed for in‐depth exploration of the development, migration, and function of targeted immune cells in zebrafish, which can potentially be extrapolated to further our understanding of cellular mechanisms in mammals.

With the tools available for zebrafish research, more studies could investigate cell size, morphology, density, and proportion prior to, during, and after intestinal inflammation, which could provide important knowledge of specific disease models.

In addition to immune cells, zebrafish represent a valuable *in vivo* model for studying the functions of specific cytokines that are absent in rodents. For instance, IL‐26, which is absent in mice but conserved in zebrafish, is produced by ILCs and is known in humans for its role in pro‐inflammatory responses, antimicrobial activity, and regulation of immune cell proliferation and differentiation [23]. Similarly, the small chemokine CXCL8 (also known as IL‐8), a potent neutrophil chemoattractant critical for directing immune cells to sites of injury, lacks a true homolog in mice and rats. Zebrafish, however, express three distinct CXCL8 homologs—Cxcl8a, Cxcl8b1, and Cxcl8b3—of which *Cxcl8a* and *Cxcl8b1* expression is induced under inflammatory conditions. These homologs have been shown to play essential roles in neutrophil recruitment and immune defense, particularly during infection and tissue injury [65, 66]. These features establish the zebrafish as a powerful alternative to rodent models for dissecting the *in vivo* functions of IL‐26, CXCL8, and related conserved cytokines involved in mucosal and innate immunity.

It is important to acknowledge that the zebrafish model has several limitations, which can challenge the direct translation of findings to mammalian systems. Notably, zebrafish lack Peyer's patches, which are critical components of the mammalian gut‐associated lymphoid tissue (GALT) and play a key role in adaptive immunity. Zebrafish do not possess an advanced compartmentalized gut structure as found in mammals and lack the crypt villus structure and submucosal plexus. Furthermore, hematopoiesis occurs in the kidneys of zebrafish, which are largely considered to be the functional counterparts to the mammalian bone marrow [67, 68]. The anatomic simplicity, while advantageous for certain studies, does not fully recapitulate the complexity and functional specialization of the mammalian gastrointestinal immune system. Some immune cell types and signaling pathways that are central to mammalian immunity are either absent / not yet identified in zebrafish. In addition, there are immune cell types that have only been described in zebrafish. As an example, metaphocytes are a unique population of tissue‐resident macrophage‐like cells identified in zebrafish. Found at all major mucosal barriers—including the intestine—metaphocytes express some phagocytic genes, but lack true phagocytic activity. Instead, they specialize in capturing external soluble antigens and transferring them to professional immune cells, thereby contributing to mucosal immune surveillance [69, 70, 71]. To date, such a cell population has not been described in mammals, suggesting that metaphocytes may represent a zebrafish‐specific adaptation for barrier immunity.

As with all model organisms, it is also important to remember that next to highly conserved mechanisms, different species might have evolved different mechanisms to counter similar threats to the host's immune system. Therefore, knowledge of research in the field of immunity in other fish species (especially those closely related to the zebrafish, for example, cyprinids) is necessary to understand the development of immunity throughout evolution [72].

Most studies have focused on the immune system during early developmental stages [73], leaving a gap in our understanding of immune processes during adulthood. Investigating the adult zebrafish immune system is particularly crucial for gaining deeper insights into the adaptive immune system, as this stage better reflects the functional complexity of immune responses observed in mature organisms.

Making use of the advantages of zebrafish to study specific immune responses during intestinal inflammation may improve our understanding of, for example, the gut–brain axis as well as neuronal, metabolic, or infectious diseases, which can all be related to imbalances within the intestinal immune system. Moreover, intestinal immune cells are influenced by external factors such as diet and the gut microbiome, which can easily be manipulated in zebrafish. Unraveling understudied cellular and molecular mechanisms could be essential for translating immune cell biology into clinical applications, enabling more precise strategies to modulate immune responses. This knowledge could pave the way for novel therapies to enhance immunity against, for example, infections and cancer, or to suppress excessive immune activation in allergic and autoimmune diseases [74]. Establishing disease models that focus on acute and chronic intestinal inflammation will also play a key role in unraveling immune cell functions and interactions in context‐dependent disease states. Future research should focus on developing cell‐type‐specific reporters, characterizing immune cell behavior prior to, during, and after inflammation, and exploring cross‐species immune mechanisms. The currently available and advanced tools underline the potential of zebrafish as a promising model for advancing our understanding of gut immunity, bridging gaps in mammalian research, and uncovering conserved and novel immune functions.

## Conflict of interests

The authors declare that they have no known competing financial interests that could have appeared to influence the work reported in this paper.

## Author contributions

AISA‐C and MM contributed equally to this work including idea, initial drafting of paper, table layout, and figure editing. All co‐authors, SM, SB, GCV, and JS, participated in the writing process of the review by editing, adding comments, and providing constructive feedback to develop the manuscript.
